# Associations of social vulnerability index with patient-reported outcomes in women treated with chemotherapy for early-stage breast cancer

**DOI:** 10.1093/oncolo/oyae311

**Published:** 2024-11-26

**Authors:** Natalie Almond, Allison M Deal, Annie Page, Kirsten A Nyrop, Hyman B Muss

**Affiliations:** School of Medicine, University of North Carolina at Chapel Hill, Chapel Hill, NC 27599-7305, United States; Lineberger Comprehensive Cancer Center, University of North Carolina at Chapel Hill, Chapel Hill, NC 27599-7305, United States; Gillings School of Global Public Health, University of North Carolina at Chapel Hill, Chapel Hill, NC 27599-7305, United States; Division of Oncology, School of Medicine, University of North Carolina at Chapel Hill, Chapel Hill, NC 27599-7305United States; Division of Oncology, School of Medicine, University of North Carolina at Chapel Hill, Chapel Hill, NC 27599-7305United States

**Keywords:** social vulnerability index, breast cancer, chemotherapy, toxicities

## Abstract

**Background:**

In a convenience sample of women scheduled for chemotherapy for early-stage breast cancer, we investigated associations of the Center for Disease Control and Prevention’s neighborhood-level social vulnerability index (SVI) with pretreatment demographics and patient-reported outcome (PRO) measures (health behavior, function and quality of life, treatment toxicities during chemotherapy).

**Methods:**

The SVI Overall score is comprised of 4 themes: socioeconomic, household composition, minority status/language, and household type/transportation, with scores ranging from 0 = lowest to 1 = highest vulnerability neighborhoods. Participant SVI scores were derived from zip codes listed in the patient’s address within the electronic medical record (EMR). Associations of study variables with SVI were evaluated using Spearman correlation for continuous variables and Kruskal–Wallis tests for categorical variables.

**Results:**

In a sample of 309 women, the mean age was 56 years (range 23-83) and 75% White. Greater vulnerability SVI Overall score was associated with lower education (*P* =.02), nonmarriage (*P* ≤.0001), higher body mass index (*P* =.03), and prechemotherapy PRO measures such as fewer self-reported walking minutes/week (*P ≤*.001), history of smoking (*P* =.02) and alcohol use (*P* < .001), depression (*P* =.01), and lower emotional social support (*P* =.008). During chemotherapy, moderate, severe, or very severe symptoms were associated with greater vulnerability SVI Overall scores for hot flashes (*P* =.03), arthralgia (*P* =.02), myalgia (*P* =.02), peripheral neuropathy (*P* =.01), edema of limbs (*P* =.04), and nausea (*P* <.001).

**Conclusions:**

SVI scores derived from addresses in the patient’s EMR can be used to generate information that adds to the patient’s social history in ways that are informative for anticipating and monitoring chemotherapy-related toxicities.

**Implications for practice**

In women with early breast cancer, this study investigates associations of a patient’s score on the Center for Disease Control and Prevention’s Social Vulnerability Index (0 = lowest vulnerability, 1 = highest vulnerability) with their prechemotherapy demographics and patient-reported outcome variables (health behavior, functional and quality of life factors, and treatment toxicities during chemotherapy). We report significant associations of higher vulnerability SVI Overall with lower education, nonmarriage, higher body mass index, lower levels of physical activity, history of smoking and alcohol use, depression, and lower emotional support, as well as higher rates of moderate, severe, or very severe patient-reported toxicities during chemotherapy (hot flashes, arthralgia, myalgia, peripheral neuropathy, edema of limbs, and nausea).

## Introduction

Social vulnerability is a concept that takes into account a wide variety of factors that may identify or clarify added risks to health and health outcomes and is central to growing interest in social determinants of health (SDOH).^1^ The U.S. Department of Health and Human Services describes SDOH as comprised of 5 domains: economic stability, education access and quality, healthcare access and quality, neighborhood and built environment, and social and community context.^1^ SDOH can provide insight into individual and population risk for disease, disability, and worst outcomes and can be especially informative for health disparities research pertaining to racially and ethnically minoritized populations and older as compared with younger patients.^1^

The Center for Disease Control and Prevention (CDC)’s social vulnerability index (SVI) is a further resource for SDOH information. Developed primarily for disaster planning and response, SVI can similarly be used to evaluate social influences on health, diagnoses, and treatment planning.^1,2^ In a recent analysis of adults with breast, lung, or colon cancer, higher mortality rates were associated with higher (greater vulnerability) SVI scores.^3^ Another study reported lower rates of breast and colon screening in more vulnerable communities.^4^ Greater vulnerability SVI scores have also been associated with increased risk for adverse outcomes following invasive treatment procedures.^5,6^ To date, health services research utilizing the SVI has been conducted mostly within large databases such as Medicare claims data.^5,6^ In the current study, we investigate the potential utility of SVI directly in the care of individual patients and in health services research as a source for data on current clinical practice.

Our research question is whether SVI scores derived from zip codes located in the electronic medical record (EMR) may provide insights into patient characteristics that could be directly relevant to cancer treatment and care decisions. In a sample of women treated with chemotherapy for early-stage breast cancer (BC), we investigate associations between SVI scores and prechemotherapy assessments and questionnaires pertaining to function and quality of life, as well as toxicities during chemotherapy.

## Methods

### Study participants

Data from 3 identical observational studies investigating home-based walking during chemotherapy for early BC—women aged 21-64 years (NCT02167932) (03/2014-11/2016), age 65 or older (NCT02328313) (10/2014-01/2019), and age 21 or older (NCT03761706) (02/2018-06/2020)—were utilized for this ancillary data analysis. There was no random or other assignment to treatment or exercise intervention. Pooled data from the three studies—with identical protocols other than age criteria—have been utilized in previously published studies.^7,8^ Studies were approved by the UNC Institutional Review Board and the UNC Lineberger Comprehensive Cancer Center Protocol Review Committee. Enrollment in the studies was from February 2014 through February 2020 (see Supplementary Appendix A for a comparison of participants in each study). Chemotherapy regimens were determined by the treating oncologists in consultation with their patients depending on tumor stage and phenotype.^9^ Daily clinic schedules were screened for potential study patients, and eligible patients were recruited and consented 1-2 weeks before the initiation of planned chemotherapy.

### Measures

#### Social vulnerability index

The SVI was developed as a public health measure to demonstrate how social vulnerabilities can affect populations during natural and manmade disasters.^2^ Using data from the U.S. Census Bureau, the SVI provides an overall value of vulnerability as well as separate values for 4 themes (domains) that comprise the SVI—Socioeconomic status (poverty, employment, income, education), Household Composition/Disability (age 65 or older, age 17 or younger, civilian with a disability, single-parent household), Minority Status/Language (racial or ethnic minority, speaks English “less than well”), and Housing Type/Transportation (multiunit structures, mobile homes, crowing, no vehicle, group quarters)—which are assessed using 15 variables (see Supplementary Appendix A).^2^ Census tracts, which can be smaller than a county, are evaluated for SVI Overall and for each of the 4 themes, with scores ranging from 0 (least vulnerable) to 1 (most vulnerable).^2^

The CDC provides guidance on how to calculate SVI scores using Geographic Identifiers (GEOIDs) designed by the Census Bureau to provide details within individual counties.^2,10^ For the current study, GEOIDs were calculated by entering the study participant’s zip code from her address in the EMR into the Census Bureau’s geocoding platform (https://geocoding.geo.census.gov/geocoder/geographies/address?form).^11^ For patients with very rural addresses, such as a PO box only, a nearby business or government building was used to identify the SVI for that address. For patients whose address in the EMR had changed since their initial entry into the EMR, their zip code at consent into one of the walking studies was used. SVI is commonly evaluated by quintile with percentile values between 0 and 1.^2,12^

- Quintile 1 (lowest vulnerability, least vulnerable)—SVI between 0 and 0.19.
- Quintile 2—SVI between 0.2 and 0.39.
- Quintile 3—SVI between 0.4 and 0.59.
- Quintile 4—SVI between 0.6 and 0.79.
- Quintile 5 (greatest vulnerability, most vulnerable)—SVI between 0.8 and 1.

#### Assessments and patient-reported outcome measures

Research staff conducted 3 assessments of study participants at baseline (before start of chemotherapy): Short Physical Performance Battery (SPPB),^13^ Timed Up and Go (TUG),^14,15^ and Blessed Orientation Memory Concentration test (BOMC).^16,17^ Also at baseline, participants completed questionnaires: Instrumental Activities of Daily Living (IADL),^18^ Mental Health (Mental Health Index/MHI)^19,20^ (subscales Depression and Anxiety), Physical Function,^21^ Social Activities,^22^ Social Support (subscales Emotional and Tangible),^23^ Quality of Life (Functional Assessment of Cancer-General/FACT-G),^24^ and Fatigue (Functional Assessment of Chronic Illness Therapy/FACIT-Fatigue).^25^ Patient questionnaires also inquired about the participant’s age, race, ethnicity, education, marital status, alcohol consumption, and prediagnosis engagement in physical activity.

#### Patient-reported symptoms

Treatment toxicity (symptom severity and symptom interference with activities of daily living) data were collected from participants at baseline (prechemotherapy) and at infusion visits throughout chemotherapy treatment (every 2-3 weeks). Symptoms pertained to 17 commonly occurring side effects of chemotherapy: fatigue, insomnia, anxiety, depression, dyspnea, peripheral neuropathy, joint pain/arthralgia, muscle pain/myalgia, abdominal pain, general pain, lymphedema/edema of the extremities, constipation, diarrhea, nausea, vomiting, mucositis, and hot flashes. The reporting formats were the patient-validated Patient-Reported Symptom Monitoring^26^ for the first 2 studies and the National Cancer Institute’s Patient-Reported Outcome (PRO)-CTCAE when it became publicly available in April 2016 for the third study.^27,28^ Both formats inquire about symptom “severity” and “interference with daily activities” with response options none, mild, moderate, severe, or very severe. Data collected from both instruments have been combined, as in our previous studies.^7,8,29,30^

#### Electronic medical record

EMR data were extracted for the following variables: height and weight for Body Mass Index (BMI), comorbidities, BC diagnosis and treatment, and clinician note descriptions of events during chemotherapy (dose delay, dose reduction, early treatment discontinuation, and hospitalization).

### Statistical considerations

In a sample of 382 participants with clinical data, 68 were excluded due to non-North Carolina addresses and 5 were excluded for missing questionnaire data. For the final sample, descriptive statistics were calculated for all study variables. Spearman correlations evaluated univariate associations between continuous patient demographic and clinical variables with SVI Overall and theme scores. Kruskal–Wallis tests were used for categorical variables. A two-tailed *P* of <.05 was considered significant. All analyses were performed with SAS statistical software (version 9.4; SAS, Cary, NC).

## Results

### Patient characteristics

In a sample of 309 women (Table 1), the mean age was 55.8 years (range 23-83), 75% White race, 15% educational level of high school or less, 58% married, 19% living alone, and 40% had obesity at diagnosis (BMI 30 or greater). BC diagnosis was 35% stage I, 45% stage II, and 21% stage III.

**Table 1.** Patient characteristics and univariate associations with SVI.

Variable	Descriptive *N* = 309 (%)	Mean SVI overall	Mean SVI socioeconomic	Mean SVI household composition	Mean SVI minority status/language	Mean SVI household type/transportation
Age—mean (SD) years, range^1^	55.8 (12.3) Range 23–83	Spearman −0.06	Spearman −0.11	Spearman −0.04	Spearman −0.04	Spearman 0.06
Race						
White	231 (75%)	0.36	0.31	0.37	0.48	0.44
Black	65 (21%)	0.53***	0.47***	0.51**	0.62**	0.54*
Other	13 (4%)	0.36	0.27	0.37	0.65	0.23
Education						
High school or less	44 (15%)	0.49*	0.44*	0.52**	0.52	0.53
More than high school	240 (85%)	0.38	0.33	0.38	0.52	0.45
Employed more than 32 hours/week						
No	176 (64%)	0.41	0.35	0.42	0.51	0.49
Yes	99 (36%)	0.39	0.34	0.38	0.54	0.43
Married						
No	118 (42%)	0.48****	0.41***	0.46*	0.58**	0.55****
Yes	166 (58%)	0.34	0.30	0.37	0.48	0.41
Living alone						
No	219 (81%)	0.40	0.35	0.41	0.50	0.45
Yes	51 (19%)	0.44	0.36	0.38	0.56	0.54*
Menopausal status at BC diagnosis						
Premenopausal	109 (36%)	0.39	0.36	0.41	0.49	0.44
Postmenopausal	197 (64%)	0.39	0.33	0.39	0.53	0.47
Body Mass Index (BMI) at BC diagnosis						
Underweight (<18.5 BMI)	3 (1%)	0.35	0.25	0.44	0.33	0.51
Normal (18.5 to <25 BMI)	64 (27%)	0.36	0.31	0.36	0.48	0.45
Overweight (25 to <30 BMI)	78 (32%)	0.39	0.32	0.40	0.52	0.46
Obese (30 or higher BMI)	97 (40%)	0.49*	0.43*	0.49*	0.58	0.52
Breast cancer stage						
1	107 (35%)	0.36	0.31	0.35	0.50	0.43
2	138 (45%)	0.40	0.34	0.41	0.52	0.48
3	63 (21%)	0.45	0.39	0.48*	0.53	0.47

Interpretation: The higher the SVI score the greater the vulnerability (range 0-1).

Bold print: * is *P*-value <.05, ** is *P-*value <.01, *** is *P-*value <.001, **** is *P-*value <.0001.

Abbreviation: SVI: social vulnerability index.

### SVI quintiles

**Figure 1.**
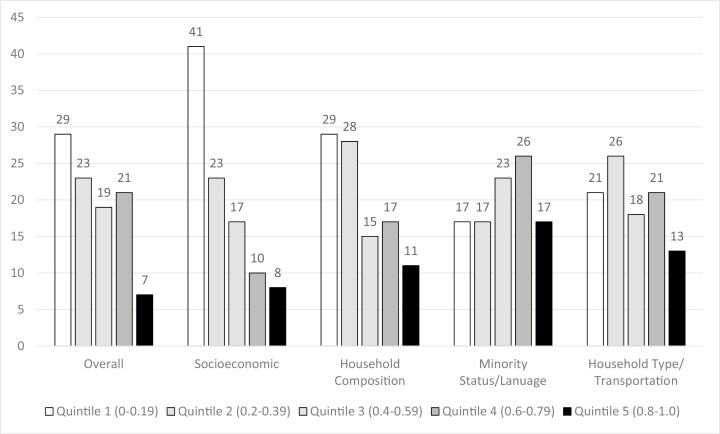
Social vulnerability index (SVI), percentage.

Women in our sample covered 224 zip codes within 41 of North Carolina’s 100 counties. Figure 1 provides an overview of SVI Overall and SVI themes by quintile numbered from 1 (least vulnerable 0-0.19) to 5 (most vulnerable 0.8-1.0). Fifty-two percent of study participants were from communities with the lowest vulnerability (quintiles 1 and 2) and 28% were from highest vulnerability communities (quintiles 4 and 5).

### Univariate associations of patient characteristics with SVI

In Table 1, significant associations (*P* ≤.05) between patient characteristics and SVI scores are identified with an asterisk (*). Nonmarried status was associated with greater vulnerability scores for SVI Overall (0.48 unmarried vs 0.34 married, *P* <.0001) and all SVI themes. High school education or less and high BMI were associated with greater vulnerability SVI Overall as well as SVI Socioeconomic and Household Composition themes. Living alone was associated with greater vulnerability scores for SVI Household Type/Transportation. Stage 3 BC was associated with greater vulnerability scores for Household Composition.

### Prechemotherapy assessments and PRO measures

Table 2 provides a summary of prechemotherapy assessments and PRO measures. The sample was relatively well-functioning, with only 3% requiring ≥14 seconds to complete the TUG test, average SPPB scores of 10.6 close to the best score of 12, and only 16% having BOMC score ≥5 signifying lower cognition. Eleven percent reported ≥1 fall in the past 6 months, 49% reported never/only a few times a week engaging in vigorous physical activity, 62% reported never smoking, and 38% reported never/almost never drinking alcohol. Twenty-five percent reported limitations in IADLs, 25% MHI Depressed, 44% MHI Anxious, 4% low emotional, and 6% low tangible social support. Mean scores for FACT-G Well-Being and FACIT-Fatigue were on the upper range, signifying higher well-being and less fatigue. Symptoms-rated moderate, severe, or very severe before chemotherapy included 30% insomnia, 25% anxiety, 16% fatigue, and 15% general pain.

**Table 2.** Univariate associations of prechemotherapy assessments and PRO measures and symptoms association with SVI.

Variable	Descriptive	Mean SVI overall	Mean SVI socioeconomic	Mean SVI household composition	Mean SVI minority status/language	Mean SVI household type/transportation
Assessments						
Timed Up and Go (TUG)						
Under 14 seconds	190 (97%)	0.41	0.34	0.43	0.52	0.47
14 seconds or more	5 (3%)	0.48	0.36	0.62	0.57	0.52
SPPB—range 0 = worst to 12 = best performance—Average (SD)	10.6 (1.8)	Spearman −0.08	Spearman −0.07	Spearman −0.14*	Spearman −0.07	Spearman −0.04
Blessed Orientation Memory Concentration Test/BOMC – lower cognition score ≥ 5						
<5	198 (84%)	0.41	0.35	0.41	0.54	0.47
≥5	37 (16%)	0.49	0.42	0.53*	0.51	0.56

Patient-reported health behavior		SVI overall	Socioeconomic	Household composition	Minority status/language	Household type/transportation
History of falls in the past 6 months						
None	245 (89%)	0.39	0.33	0.40	0.55	0.46
1 or more	29 (11%)	0.43	0.42	0.45	0.50	0.46
Self-reported history of walking minutes per week—Average (SD)^a^	129 (140)	Spearman −0.21***	Spearman −0.23****	Spearman −0.26****	Spearman −0.14*	Spearman −0.08
Vigorous physical activity						
Never, a few times a week	122 (49%)	0.42*	0.37	0.42	0.55	0.46*
1-2 times a week	58 (21%)	0.43*	0.36	0.43	0.50	0.52*
3 or more times a week	81 (30%)	0.32*	0.27	0.34	0.47	0.41*
Smoking history						
Never	168 (62%)	0.37*	0.32*	0.37*	0.49	0.44
Past smoker	83 (31%)	0.43*	0.38*	0.40*	0.55	0.50
Current smoker	21 (8%)	0.54*	0.45*	0.56*	0.60	0.53
Alcohol Use						
No, almost never	105 (38%)	0.33***	0.28***	0.33***	0.47**	0.42
Yes	171 (62%)	0.43***	0.38***	0.45***	0.55**	0.49

PROs measures		SVI overall	Socioeconomic	Household composition	Minority status/language	Household type/transportation
Instrumental Activities of Daily Living/IADL—range 0–14						
14 = no limitations	232 (75%)	0.39	0.33	0.39	0.51	0.46
<14 = limitations	77 (25%)	0.43	0.38	0.43	0.54	0.48
Mental Health Index/MHI—range 0–43						
Depressed score ≥ 12						
Not depressed	202 (75%)	0.37*	0.32*	0.39	0.50*	0.44**
Depressed	66 (25%)	0.47*	0.41*	0.42	0.58*	0.54**
Mental Health Index/MHI—range 0–20						
Anxious score ≥ 6						
Not anxious	154 (56%)	0.38	0.32	0.40	0.51	0.44
Anxious	123 (44%)	0.43	0.37	0.40	0.53	0.50
Social Support-Emotional – range 0–100						
<50 low support	8 (4%)	0.66**	0.55*	0.57	0.79**	0.64
≥50 high support	181 (96%)	0.39**	0.33*	0.41	0.50**	0.46
Social Support-Tangible (range 0–100)						
<50 low support	12 (6%)*	0.55*	0.43	0.46	0.68*	0.65*
≥50 high support	177 (94%)	0.39*	0.33	0.41	0.50*	0.45*
Functional Assessment of Cancer Therapy/FACT-General (higher score = higher well-being)—Average (SD)						
Physical well-being (range 0–28)	24.8 (3.9)	Spearman −0.0	Spearman −0.06	Spearman −0.09	Spearman −0.00	Spearman −0.01
Social/family wellbeing (range 0–28)	24.8 (4.5)	Spearman −0.08	Spearman −0.09	Spearman −0.03	Spearman −0.06	Spearman −0.05
Emotional wellbeing (range 0–24)	18.9 (3.9)	Spearman 0.01	Spearman −0.03	Spearman 0.02	Spearman 0.04	Spearman 0.03
Functional wellbeing (range 0–28)	20.7 (5.6)	Spearman −0.03	Spearman −0.07	Spearman −0.02	Spearman −0.02	Spearman −0.05
Functional Assessment of Chronic Illness Therapy/FACIT-Fatigue (reverse scored so that higher score = less fatigue) (range 0–160)—Average (SD)	132 (21)	Spearman −0.05	Spearman −0.08	Spearman −0.05	Spearman −0.03	Spearman −0.03

PROs symptoms (rated moderate, severe, or very severe [MSVS]) before chemotherapy initiation		SVI overall	Socioeconomic	Household composition	Minority status/language	Household type/transportation
Fatigue						
Yes	47 (16%)	0.40	0.35	0.45	0.49	0.44
No	262 (85%)	0.40	0.34	0.39	0.52	0.46
Anxiety						
Yes	76 (25%)	0.40	0.33	0.39	0.50	0.48
No	233 (75%)	0.40	0.35	0.41	0.52	0.45
Depression						
Yes	48 (16%)	0.36	0.31	0.34	0.48	0.46
No	261 (84%)	0.41	0.35	0.41	0.52	0.45
Insomnia						
Yes	93 (30%)	0.43	0.38	0.45*	0.50	0.48
No	216 (70%)	0.38	0.33	0.38*	0.52	0.45
Hot flashes						
Yes	28 (9%)	0.42	0.35	0.41	0.54	0.48
No	281 (91%)	0.40	0.34	0.40	0.51	0.46
Dyspnea						
Yes	7 (2%)	0.27	0.25	0.44	0.38	0.24*
No	302 (98%)	0.40	0.35	0.40	0.52	0.47*
Aching joints/arthralgia						
Yes	32 (10%)	0.52*	0.45*	0.54**	0.60*	0.51
No	277 (90%)	0.38*	0.33*	0.39**	0.51*	0.46
Aching muscles/myalgia						
Yes	28 (9%)	0.48	0.43	0.50*	0.58	0.49
No	281 (91%)	0.39	0.33	0.39*	0.51	0.46
Peripheral neuropathy						
Yes	9 (3%)	0.50	0.44	0.53	0.49	0.57
No	300 (97%)	0.40	0.34	0.40	0.52	0.46
Edema limbs						
Yes	6 (2%)	0.40	0.39	0.51	0.36	0.41
No	303 (98%)	0.40	0.34	0.40	0.52	0.46
Abdominal pain						
Yes	10 (3%)	0.38	0.33	0.48	0.43	0.43
No	299 (97%)	0.40	0.34	0.40	0.52	0.46
General pain						
Yes	45 (15%)	0.43	0.39	0.45	0.51	0.46
No	264 (85%)	0.39	0.34	0.39	0.52	0.46
Constipation						
Yes	31 (10%)	0.50*	0.43*	0.51*	0.58	0.53
No	278 (90%)	0.39*	0.33*	0.39*	0.51	0.45
Diarrhea						
Yes	33 (11%)	0.38	0.32	0.42	0.47	0.44
No	276 (89%)	0.40	0.35	0.40	0.52	0.46
Nausea						
Yes	7 (2%)	0.39	0.32	0.32	0.63	0.48
No	302 (98%)	0.40	0.34	0.40	0.51	0.46
Vomiting						
Yes	2 (1%)	0.14	0.08	0.08*	0.37	0.37
No	307 (99%)	0.40	0.34	0.40*	0.52	0.46
Mucositis oral						
Yes	7 (2%)	0.34	0.29	0.34	0.52	0.38
No	302 (98%)	0.40	0.34	0.40	0.52	0.46

Interpretation: The higher the SVI, the greater the vulnerability (range 0-1).

Bold print: * is *P*-value <.05, ** is *P*-value <.01, *** is *P*-value <.001, **** is *P-*value <.0001.

^a^For continuous variables, instead of reporting mean SVI values by group, a Spearman Correlation Coefficient is reported.

Abbreviations: PRO: Patient-Reported Outcome; SPPB: Short Physical Performance Battery; BOMC: Blessed Orientation Memory Concentration Test; MHI: Mental Health Index; SVI: Social Vulnerability Index; IADL: Instrumental Activities of Daily Living; FACT: Functional Assessment of Cancer Therapy; FACIT: Functional Assessment of Chronic Illness Therapy.

### Associations of prechemotherapy assessments and PRO measures with SVI

For SVI Overall, lowest vulnerability scores were associated with higher rates of prechemotherapy walking minutes/week (*P* <.001), engaging in vigorous activity 3 or more times a week (*P* =.02), never smoking (*P* =.02), and low/no alcohol use (*P* <.001). Higher self-reported walking before chemotherapy was also associated with the lowest SVI scores for SES, Household Composition, and Minority Status/Language. For symptoms-rated moderate, severe, or very severe (MSVS) before chemotherapy initiation, greater vulnerability SVI Overall and theme scores were associated with MSVS aching of joints/arthralgia and constipation.

### Patient-reported toxicities during chemotherapy

Table 3 provides an overview of patient-reported symptom “severity” and “symptom interference with daily living” during chemotherapy. The highest proportions of symptoms-rated MSVS severity were fatigue (67%), insomnia (56%), diarrhea (47%), anxiety (42%), and general pain (41%). For symptom interference with activities of daily living, the highest proportions-rated MSVS were fatigue (61%), insomnia (38%), diarrhea (38%), nausea (34%), and general pain (32%). Regarding events during chemotherapy, 15% were hospitalized, 37% had dose reduction, 18% had dose delay, and 16% had early treatment discontinuation.

**Table 3.** Associations of patient-reported toxicities and events during chemotherapy with SVI.

Variable	Symptom-rated MSVS^a^ (%)	Mean SVI overall	Mean SVI socioeconomic	Mean SVI household composition	Mean SVI minority status/language	Mean SVI household type/transportation
Symptom severity						
Fatigue						
Yes	207 (67%)	0.40	0.35	0.41	0.53	0.47
No	102 (33%)	0.39	0.33	0.39	0.50	0.44
Anxiety						
Yes	129 (42%)	0.41	0.34	0.40	0.54	0.48
No	180 (58%)	0.39	0.34	0.41	0.50	0.45
Depression						
Yes	96 (31%)	0.41	0.36	0.36	0.51	0.48
No	213 (69%)	0.42	0.36	0.42	0.52	0.45
Insomnia						
Yes	173 (56%)	0.41	0.35	0.41	0.53	0.47
No	136 (44%)	0.39	0.33	0.39	0.51	0.45
Hot flashes						
Yes	106 (34%)	0.44*	0.38*	0.44	0.55	0.48
No	203 (66%)	0.37*	0.32*	0.38	0.50	0.45
Dyspnea						
Yes	54 (17%)	0.39	0.34	0.42	0.52	0.44
No	255 (83%)	0.40	0.34	0.40	0.52	0.46
Aching joints/arthralgia						
Yes	112 (36%)	0.45*	0.38	0.46**	0.55*	0.50
No	197 (64%)	0.37*	0.32	0.37**	0.50*	0.44
Aching muscles/myalgia						
Yes	115 (37%)	0.44*	0.37	0.44*	0.56*	0.51**
No	194 (63%)	0.37*	0.33	0.38*	0.49*	0.43**
Peripheral neuropathy						
Yes	89 (29%)	0.46*	0.41**	0.44	0.57*	0.50
No	220 (71%)	0.37*	0.32**	0.39	0.50*	0.44
Edema limbs						
Yes	57 (18%)	0.47*	0.42*	0.49*	0.54	0.51
No	252 (82%)	0.38*	0.32*	0.38*	0.51	0.45
Abdominal pain						
Yes	68 (22%)	0.43	0.37	0.45	0.51	0.48
No	241 (78%)	0.39	0.33	0.39	0.52	0.46
General pain						
Yes	128 (41%)	0.40	0.34	0.42	0.52	0.45
No	181 (59%)	0.39	0.34	0.39	0.51	0.47
Constipation						
Yes	103 (33%)	0.44	0.37	0.43	0.56*	0.48
No	206 (67%)	0.38	0.33	0.39	0.50*	0.45
Diarrhea						
Yes	144 (47%)	0.41	0.36	0.42	0.52	0.47
No	165 (53%)	0.38	0.33	0.39	0.51	0.45
Nausea						
Yes	97 (31%)	0.48***	0.42**	**0.44**	0.60****	0.51*
No	212 (69%)	0.36***	0.31**	**0.38**	0.48****	0.44*
Vomiting						
Yes	23 (7%)	0.48	0.42	0.46	0.59	0.50
No	286 (93%)	0.39	0.34	0.40	0.51	0.46
Mucositis oral						
Yes	68 (22%)	0.44	0.38	0.42	0.55	0.50
No	241 (78%)	0.39	0.33	0.40	0.51	0.45

Interference with daily activities						
Fatigue						
Yes	189 (61%)	0.42	0.37	0.41	0.53	0.48
No	120 (39%)	0.37	0.31	0.39	0.49	0.44
Anxiety						
Yes	70 (23%)	0.42	0.36	0.41	0.56	0.49
No	239 (77%)	0.39	0.34	0.40	0.50	0.45
Depression						
Yes	55 (18%)	0.44	0.40	0.40	0.51	0.51
No	254 (82%)	0.39	0.33	0.40	0.52	0.45
Insomnia						
Yes	118 (38%)	0.41	0.37	0.41	0.52	0.46
No	191 (62%)	0.39	0.33	0.40	0.52	0.46
Hot flashes						
Yes	83 (27%)	0.44	0.40**	0.41	0.55	0.49
No	226 (73%)	0.38	0.32**	0.40	0.51	0.45
Dyspnea						
Yes	53 (17%)	0.44	0.40	0.45	0.55	0.47
No	256 (83%)	0.39	0.33	0.39	0.51	0.46
Aching joints/arthralgia						
Yes	85 (28%)	0.46*	0.39*	0.47**	0.54	0.51*
No	224 (72%)	0.37*	0.33*	0.38**	0.51	0.44*
Aching muscles/myalgia						
Yes	84 (27%)	0.46**	0.40**	0.44	0.56	0.51*
No	225 (73%)	0.38**	0.32**	0.39	0.50	0.44*
Peripheral neuropathy						
Yes	55 (18%)	0.50**	0.47***	0.46	0.59*	0.51
No	254 (82%)	0.38**	0.32***	0.39	0.50*	0.45
Edema limbs						
Yes	37 (12%)	0.52**	0.47**	0.54**	0.57	0.53
No	272 (88%)	0.38**	0.33**	0.38**	0.51	0.45
Abdominal pain						
Yes	48 (16%)	0.42	0.36	0.45	0.51	0.47
No	261 (84%)	0.39	0.34	0.39	0.52	0.46
General pain						
Yes	98 (32%)	0.44	0.39	0.45*	0.52	0.47
No	211 (68%)	0.38	0.32	0.38*	0.52	0.45
Constipation						
Yes	69 (22%)	0.45	0.39	0.44	0.54	0.52
No	240 (78%)	0.38	0.33	0.39	0.51	0.44
Diarrhea						
Yes	117 (38%)	0.43	0.38	0.42	0.53	0.48
No	192 (62%)	0.38	0.32	0.39	0.51	0.45
Nausea						
Yes	106 (34%)	0.43	0.37	0.42	0.55	0.48
No	203 (66%)	0.38	0.33	0.39	0.50	0.45
Vomiting						
Yes	28 (9%)	0.45	0.39	0.47	0.52	0.49
No	281 (91%)	0.39	0.34	0.40	0.52	0.46
Mucositis oral						
Yes	31 (10%)	0.46	0.38	0.45	0.51	0.53
No	278 (90%)	0.39	0.34	0.40	0.52	0.45

Events during chemotherapy						
Hospitalized						
No	262 (85%)	0.39	0.34	0.40	0.52	0.46
Yes	47 (15%)	0.42	0.36	0.44	0.52	0.48
Dose reduction						
No	196 (63%)	0.39	0.33	0.40	0.51	0.45
Yes	113 (37%)	0.42	0.36	0.41	0.53	0.48
Dose delay						
No	253 (82%)	0.39	0.34	0.40	0.50*	0.46
Yes	56 (18%)	0.43	0.37	0.39	0.58*	0.49
Early treatment discontinuation						
No	259 (84%)	0.39	0.33	0.39	0.51	0.45
Yes	59 (16%)	0.46	0.39	0.47	0.57	0.52

^a^Symptom-rated moderate, severe, or very severe (MSVS) at any time during chemotherapy.

Interpretation: The higher the SVI, the greater the vulnerability (range 0–1).

Bold print: * is *P*-value <.05, ** is *P*-value <.01, *** is *P*-value <.001, **** is *P*-value <.0001.

Abbreviation: SVI: social vulnerability index.

### Patient-reported toxicities during chemotherapy and associations with SVI

In Table 3, higher vulnerability SVI Overall scores were associated with MSVS symptom severity for hot flashes (*P* =.03), aching joints/arthralgia (*P* =.02), aching muscles/myalgia (*P* =.02), peripheral neuropathy (*P* =.01), edema of limbs (*P* =.04), and nausea (*P* <.001). Similarly, higher vulnerability SVI Overall scores were associated with MSVS symptom interference with daily activity for arthralgia (*P* =.01), myalgia (*P* =.01), peripheral neuropathy (*P* =.009), and edema of limbs (*P* =.003). Dose delay was associated with more vulnerable SVI Minority Status/Language (*P* =.03).

## Discussion

The overall aim of our study was to explore whether the CDC’s SVI can provide patient information of use to clinicians treating women with early breast who were scheduled to receive chemotherapy. In our sample, higher vulnerability SVI scores were associated with high school education or less, not being married, higher BMI, being less physically active, current smoker, alcohol use, depression, lower social support, and lower social/family well-being. The SVI Overall and theme scores, in essence, provide a snapshot of social history information specific to the individual patient. Higher vulnerability scores were also associated with moderate, severe, or very severe treatment toxicities during chemotherapy, again providing a snapshot for clinicians regarding specific patients who may need additional monitoring and early interventions during active treatment and beyond.

Our study adds to the health services literature pertaining to SVI as a potential tool for risk stratification in health services research pertaining to adults with cancer.^31^ In a recent scoping review, 31 studies analyzing associations with SVI were identified and included a wide variety of solid tumor and hematologic cancers; however, none pertaining to BC. Fifty-five percent of the studies evaluated associations of SVI with surgery or transplant decisions and outcomes. Others pertained to SVI associations with mortality, incidence of cancer, disease subtype and stage, physical frailty, participation in clinical trials, virtual clinic visits, and comorbidities. No studies to date have pertained to the associations explored in our study.

We also report significant associations between SVI scores and health behavior at BC diagnosis—walking, vigorous physical activity, smoking history, and alcohol use—again, variables often included in the patient history. And, we report significant associations between SVI and PRO measures—Mental Health Index-Depression and social support emotional and tangible—that could be explored using standard-of-care inquiries about depression and social circumstances. All of these variables provide a context for the observed associations of SVI with symptom experience before and during chemotherapy, as well as how these symptoms interfere with the patient’s activities of daily living. Our SVI findings provide added context for our prior studies in which we have reported symptom experiences between patients with high as compared with normal BMI,^32^ Black and White patients,^7^ and younger as compared with older patients.^8^ To the extent it is possible for routinely collected zip codes in the EMR to be programmed to derive SVI scores, these scores may provide information that encourages additional questions during the patient’s history and special monitoring throughout the cancer care continuum.

A limitation of our study is that our data are derived from a single, university-affiliated healthcare institution and may not be representative of early BC patients treated in community-based cancer centers. However, our data did show similar trends as reported in published research reporting clinical SVI associations in large datasets.^5,6^ Second, our convenience sample was derived from participants in intervention studies and may not reflect the general population of patients. Lastly, 52% of our sample was from least vulnerable communities (SVI Overall quintiles 1 and 2) as compared with 28% from most vulnerable (quintiles 4 and 5), which again may not be representative of the general population of women with BC.

A strength of our study is the diversity in our patient population, which includes 23% who self-identified as Black. Our sample also includes considerable variability in SVI scores, allowing us to explore associations with common clinical metrics (demographics and BC diagnosis) but also with prechemotherapy assessments and questionnaires, and during treatment symptom reports.

Our findings provide promising support for the potential use of SVI as an EMR-based resource for patient history and SDOH to inform patient care. SVI could be used as a patient-history resource for clinicians interested in considering a patient’s social vulnerability in terms that may be relevant to their care and follow-up but also knowing that every patient’s lived experience is unique and SVI is just an aggregated measure. This could become a viable option if the EMR were to include an automatic SVI calculation from the patient’s zip code, highlighted as a “one click” link to the SVI Overall score and added clicks for SVI themes. While not replacing a thorough social history, SVI scores could be useful for newly arrived patients with limited medical records or when history taking is hampered at the time a patient is admitted or seen in clinic. Within the EMR, the first click would include a one-sentence reminder that 1 = least vulnerable to 5 = most vulnerable. After primary treatment, SVI scores may also be informative for identifying patients whose higher vulnerability scores may suggest difficulties with compliance with endocrine treatments that need to be taken for 5-10 years.^33^

SVI scores embedded in EMRs can also be a valuable adjunct to detailed clinical data for studies in health services research.^34–36^ This might include, for example, a study of the relationship between SVI scores and BC treatment outcomes, especially in racial and ethnic disparities research. SVI could also be valuable in the clinical trial setting, to identify patients who may be at high risk for treatment toxicities and warrant targeted symptom monitoring and management. For all of these reasons, embedding SVI scores within the EMRs, with easy one-click access for clinicians, warrants further exploration as a quality improvement tool.

## Supplementary Material

oyae311_suppl_Supplementary_Appendix_A

## Data Availability

Requests for access data can be submitted to the senior author, Kirsten A. Nyrop, PhD, at Kirsten_nyrop@med.unc.edu.

## Previous presentation

This work has been previously presented at the Presidential Poster Session of the Annual Scientific Meeting of the American Geriatrics Society on May 4, 2023. Additional presentations include the 2022 UNC Geriatric Oncology Research Symposium: Cancer and Aging on December 2, 2022 and the 55th Annual John B. Graham UNC Student Research Day on November 11, 2022.
