# Supplementary material for: Associations of social vulnerability index with patient-reported outcomes in women treated with chemotherapy for early-stage breast cancer
Source: Oncologist. 2024 Nov 26;30(7):oyae311. doi: 10.1093/oncolo/oyae311 (PMC12311274; doi:10.1093/oncolo/oyae311)

**Supplemental SVI Appendix A**

**Centers for Disease Control and Prevention (CDC) Defined SVI measures**


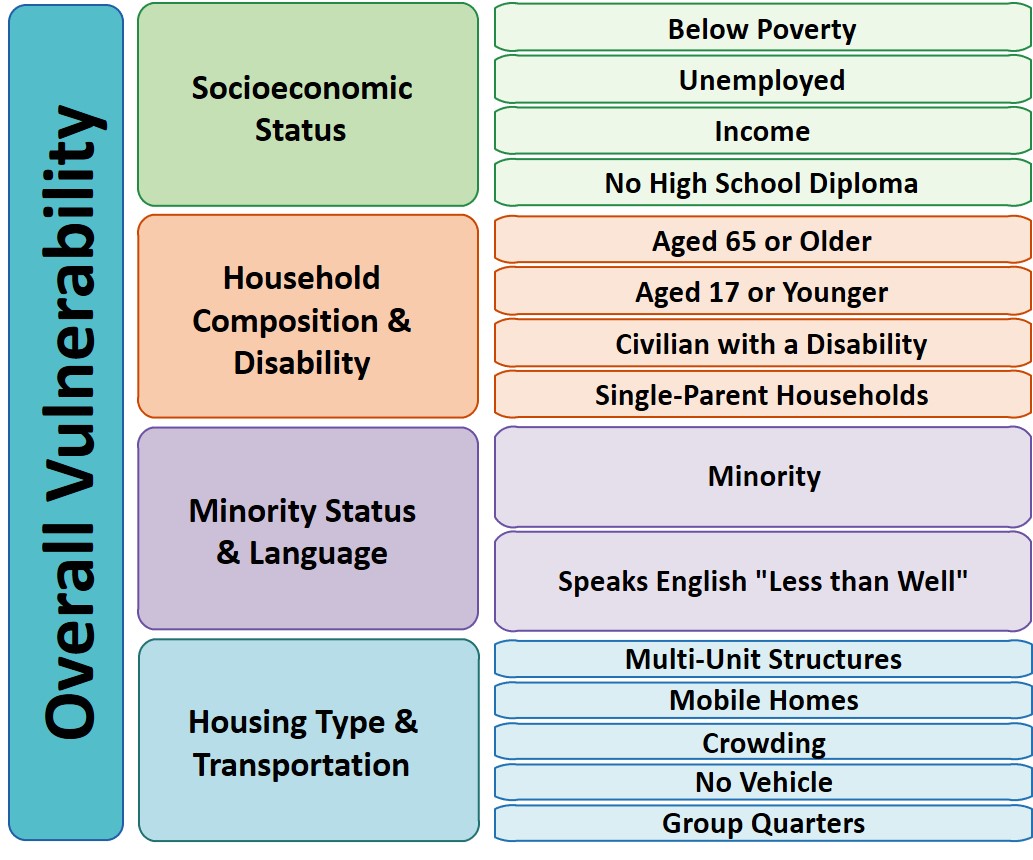

Supplement: oyae311_suppl_Supplementary_Appendix_A [file oyae311_suppl_supplementary_appendix_a.docx]
